# Gastric Dilatation-Volvulus in Dogs: Analysis of 130 Cases in a Single Institution

**DOI:** 10.3390/ani15040579

**Published:** 2025-02-18

**Authors:** Matteo Olimpo, Sabrina Cillari, Erica Ilaria Ferraris, Davide Giacobino, Paolo Savarino, Lisa Adele Piras, Greta Martinelli, Emanuela Maria Morello

**Affiliations:** Department of Veterinary Sciences, University of Turin, 10095 Grugliasco, TO, Italy; matteo.olimpo@unito.it (M.O.); sabrina.cillari@edu.unito.it (S.C.); ericailaria.ferraris@unito.it (E.I.F.); davide.giacobino@unito.it (D.G.); paolo.savarino@unito.it (P.S.); lisa.piras@unito.it (L.A.P.); greta.martinelli@unito.it (G.M.)

**Keywords:** GDV, dog, gastropexy, splenectomy, prognosis

## Abstract

Gastric dilatation-volvulus (GDV) syndrome is a life-threatening emergency and its physiopathology and treatment have been studied for decades. Despite ongoing research, the mortality rate is still high. The aims of this study are to describe the treatment and outcome of GDV patients treated from 2011 to 2024 at the veterinary teaching hospital of Grugliasco (Turin, Italy); to analyze risk and prognostic factors, comparing the obtained data with current literature; and to evaluate how patients’ management has changed over the years. The study included 130 dogs with a confirmed GDV diagnosis that underwent surgery. The data were extracted from the digital and hardcopy clinical record, combined with the imaging diagnostic software and an interview submitted to the dogs’ owners. The analysis showed the predominance (25.38%) of German Shepherd dogs, as well as of males (59.25%); among the latter, intact dogs were most represented (53.1%). Age between 5–10 years was most frequent in the examined population (54.69%). The surgical technique went through changes during the examined period: the belt loop has been abandoned in favor of the incisional gastropexy. The survival rate of GDV surgically treated dogs was 86.4%. Lactate blood concentration and splenectomy were not assessed as relevant prognostic factors.

## Simple Summary

This study analyzes the risk and prognostic factors, treatment modality and outcome of dogs with gastric dilatation-volvulus (GDV) syndrome that were encountered in a single veterinary teaching hospital. Information about patient anamnesis, treatment and follow up were collected from the management software and clinical charts. The main findings range from the prevalence of the syndrome in different breeds to surgical techniques, perioperative complications and survival. While the results about age, weight, preexisting pathologies and mortality are comparable with those of previous studies, the findings about prognostic factors, such as lactate concentration and splenectomy are not aligned. Moreover, a shift in the surgical techniques and in the medical protocols during the years was noted.

## 1. Introduction

Gastric dilatation-volvulus (GDV) syndrome is characterized by the rotation of the stomach around its own axis, compromising vascularization both locally and systemically. This can cause problems, such as gastric wall necrosis, impaired cardiac function, and even lead to the death of the patient [1]. GDV requires surgical procedures such as stomach detorsion, evaluation and removal of any necrotic areas/organs, gastropexy.

The current literature identifies several risk factors, such as breed, size, age, weight, pre-existing gastrointestinal conditions, dietary habits (intake of dry food or large amounts of liquids), and activity after meals, which appear to predispose to the development of this condition [1,2,3]. Similarly, some studies recognize prognostic factors, such as blood lactate levels or the presence of inflammatory cytokines, as well as the need to perform a splenectomy and a partial gastrectomy, hypotension, peritonitis, sepsis and more [4,5,6]. Various surgical gastropexy techniques and medical therapies are described for the treatment of GDV patients, but no uniform consensus exists on the matter [7,8]. Reported mortality rates for this condition range between 10% and 30% [1,6,9].

GDV was chosen as the subject of this study due to its clinical significance and to the complexity of its management. Although extensive research exists on the topic, the available data are often different among studies. Surgical techniques, as well as therapeutic strategies, are continuously evolving.

This retrospective observational study focuses on cases of gastric dilatation-volvulus (GDV) treated at the veterinary teaching hospital (VTH) of the University of Turin, in Grugliasco (Italy), analyzing data related to patients. The primary objective of this study is to describe the treatment and outcome of GDV patients from 2011 to 2024 at the VTH of Grugliasco (Turin, Italy) and to analyze risk and prognostic factors, comparing the obtained data with current literature. Another aim was to determine whether patients’ management had changed over the years.

## 2. Materials and Methods

This study was conducted at the emergency service (ES) of the VTH of the University of Turin, on dogs with GDV between August 2011 and June 2024. All patients with a radiologically confirmed diagnosis of GDV that underwent surgical treatment were included.

The data were collected through the review of medical records (ES, anesthesiologic, surgical and discharge report). Dogs’ admission time to ES, signalment (breed, sex, age, weight) and anamnestic data (comorbidities, feeding habits) were also retrieved. Respiratory and heart rate, body temperature, mucous membrane and hemodynamic status, state of consciousness, presence of abdominal effusion, blood test results (complete blood count, biochemical and gas analysis) were obtained from ES medical reports.

Records on the patients’ hospitalization period with information on the medical therapies administered and any complications that occurred before discharge were also reviewed. Complications were classified as follows: pre- and intraoperative complications, postoperative complications, and post-discharge complications. To complete information regarding the patients’ eating habits and the period following discharge, dogs’ owners were asked to voluntarily complete a questionnaire (see File S1 in the Supplementary Materials).

The radiographs taken at the time of dog admission to the emergency service to confirm the GDV syndrome and which were saved on MedDreamVET 7.1.1 imaging diagnostic software (PACS) (Softneta, Kaunas, Lithuania) were also reviewed by the authors (SC, EIF). To determine the arrival time of the patients at the emergency service, the time recorded in the MedDreamVET 7.1.1 diagnostic imaging software was used. This time is logged when the radiographs are performed. Therefore, it represents the most objective data available.

Statistical analysis was performed using RCommander software, version 4.4.1 (R Foundation, Vienna, Austria), and Microsoft Excel version 16.37 (Microsoft Corporation—Redmond, WA, USA). Descriptive statistical analyses of the variables analyzed were carried out, evaluating the main measures of central tendency and their dispersion measures. For this purpose, the Lilliefors normality test was used to assess the normality of the data regarding age. Single-sample T-tests were performed to compare the sample mean with the population mean; reference means from two different studies reported in the literature were used (from Brockman et al., 1995 [10] and from Buber et al., 2007 [11]). A chi-square test (χ^2^) was then conducted to verify whether there was a statistically significant difference among the different ranges, i.e., whether the category data distribution was uniform. A significance level of 0.05 was set for the tests.

Values of lactates equal to or lower than 2.5 mmol/L were considered normal, while values greater than 2.5 mmol/L were considered elevated [9]. Contingency tables were created to associate the lactate levels, categorized as “normal” or “elevated”, with the cause of death, which was categorized as “related to GDV” or “not related to GDV”. Based on a contingency table, a chi-square test was performed to assess the presence of a statistically significant difference between performing an incisional or a belt loop gastropexy, between performing or not a splenectomy and gastrectomy, and between lactate levels, all related to death cases.

## 3. Results

A total of 173 dogs with GDV syndrome were referred to the ES of VTH from 2011 to 2024. Owners of 43 dogs refused the surgical treatment and their dogs were excluded from the present study, therefore a total of 130 dogs with a surgically treated GDV syndrome were included. It was possible to review records of all the patients included in the study, although not all of them were complete.

### 3.1. Signalment

The first group of data analyzed was related to signalment. As only cases of patients with confirmed GDV were included in the study, it was not possible to conduct an incidence study. Therefore, a prevalence study was carried out regarding the breeds and sex of the examined dogs, and the distribution of the patients’ age and weight was analyzed.

#### 3.1.1. Breed

**Figure 1:**
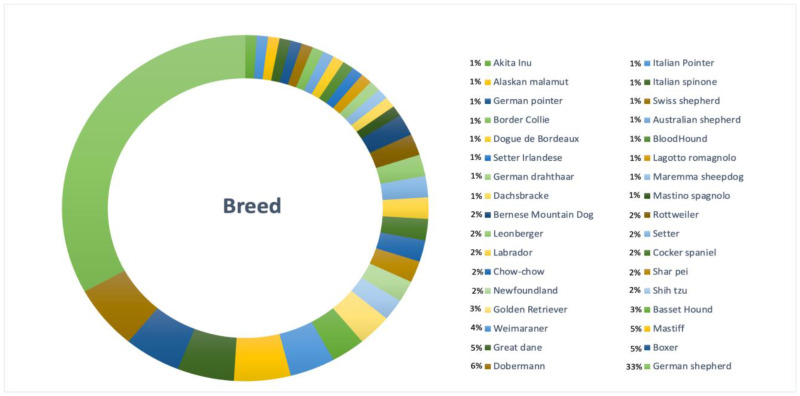
Distribution of breeds in the study sample.

Among the 130 cases included, 35 breeds were identified. Twenty-three percent (30/130) were mixed-breed dogs (see Figure 1).

To evaluate purebred prevalence, mixed-breed dogs were excluded from further analyses. The analysis revealed that the most represented purebred was the German Shepherd (25.38%; 33/130), with a prevalence among purebreds of 33% (33/100). Dobermann was the second most represented purebred (4.62%; 6/130), with a prevalence of 6% (6/100). Great Dane, Boxer and Mastiff breeds contributed to the total sample for 3.85% each (5/130); their prevalence was 5% (5/100) each.

#### 3.1.2. Sex

The data about dogs’ sex, obtained from medical records, were grouped into four categories: intact females (IF), spayed females (SF), intact males (IM), and neutered males (NM). The analysis revealed that most of the dogs included in the study were IM (69 dogs, 53.1%); 8 dogs were NM (6.15%). IF represented the second-largest group (35 dogs, 26.9%) and 18 patients were SF (13.8%).

#### 3.1.3. Age

For the analysis of the study sample, age was approximated to the nearest year. A Lilliefors normality test was performed, which rejected the hypothesis of the normality of the data (*p*-value: 0.0394). The mean age was 8.73 years, and the standard deviation (SD) 3.61 years. The following results were obtained from the T-test that compares the sample mean with two chosen population means:

- Difference with population mean A—7.5 years (from Brockman et al., 1995 [10]): statistically significant difference, *p*-value < 0.001.
- Difference with population mean B—8 years (from Buber et al., 2007 [11]): statistically significant difference, *p*-value < 0.05.

Then the data were grouped into three age ranges to observe any prevalence within a specific age group. The data were divided into the following age ranges, with their respective frequencies and percentages:

- Under 5 years: 17 occurrences, representing 13.28% of the total.
- Between 5 and 10 years: 70 occurrences, representing 54.69% of the total.
- More than 10 years: 41 occurrences, representing 32.03% of the total.

This grouping shows a high frequency in the central group (5–10 years). The chi-square χ^2^ test yielded a value of 33.02, with a *p*-value < 0.001. This result indicates a statistically significant difference between the observed distribution of the categories and an expected uniform distribution.

#### 3.1.4. Weight

The mean weight was 34.8 kg (range: 10–80 kg). To observe its distribution, the data were grouped into 10 kg intervals (from 10 to 80 kg). The largest group falls within the 31–40 kg range, with 55/130 cases.

### 3.2. Anamnesis

Data regarding the past medical history were collected from clinical records and were available only for 51 patients. The results are reported as follows: 11 dogs had gastrointestinal conditions (11/51, 21.6%), including gastritis (2/11), frequent episodes of diarrhea (1/11), food allergies (5/11), previous episodes of dilation (1/11), megaesophagus (1/11), paralytic ileus, and megacolon (1/11). Two dogs had hypothyroidism (2/51) and four dogs had cardiopathy (4/51). Six dogs had other conditions, including leishmaniasis (1/6), autoimmune diseases (1/6), neoplastic conditions (1/6), laryngeal paralysis (1/6), intervertebral disc hernia (1/6), and vestibular syndrome (1/6).

Fifty-one owners answered the questionnaire, though not all were fully completed. Data on the type of diet were obtained for 48 dogs and grouped into the categories of “dry food”, “wet food”, and “mixed diet”. The “mixed diet” category included homemade diets as well as commercial dry diets supplemented with homemade food or commercial wet food. Commercial dry food was used by 35 owners, and a mixed diet was used by 13 owners. In no cases was the diet exclusively wet food. Regarding feeding habits, data were collected about the number of meals per day given to the patients in the days leading up to the GDV episode. A tendency towards feeding two meals per day was observed, with a total of 36/48 cases, while only a minority of cases received one (6/48) or three meals (6/48) per day. From the telephone interviews, 6 owners out of 51 reported that, in the hours before the onset of GDV, the patients had not eaten but had instead ingested large amounts of water, but the amount of water was not quantified.

### 3.3. Time Distribution

The data collection covered the years from 2011 to 2024. To visualize the distribution of cases over the years, a histogram was created (see Figure S1A in the Supplementary Material). No differences were observed across years, nor does there appear to be a pattern in the occurrence of GDV over the months or seasons. To evaluate a seasonal trend, the same type of graph was created by grouping the GDV occurrences by quarter (see Figure S1B in the Supplementary Material).

### 3.4. Presentation of the Patient at the Emergency Service

The arrival time of the patients at VTH and the time between the onset of symptoms and hospitalization have been evaluated. Data regarding the diagnostic tests performed were also examined.

#### 3.4.1. Arrival Time

The data obtained for 120/130 dogs were examined and grouped into three-time ranges: morning (from 06:01 AM to 2:00 PM); afternoon (from 2:01 PM to 10:00 PM); and night (from 10:01 PM to 6:00 AM).

The following frequencies were obtained: the arrival time was in the morning, in the afternoon and in the night for 25/120, 36/120, 59/120 dogs, respectively.

To determine the onset time of the GDV symptoms, the descriptions provided by the owners through the questionnaire were used. The time difference between the one indicated by the owners and the time when the radiographic exam was taken was then calculated. In most cases, the patients were brought to the emergency service within 3 h of the onset of symptoms. In four cases, patients were hospitalized more than 24 h after the owners noticed the symptoms.

#### 3.4.2. Clinical Evaluation

The data from the ES medical report were grouped and reported, using Excel spreadsheets, in pivot tables. Table 1 was then created to summarize the data and visualize the abnormalities found in the patients.

### 3.5. Surgical Techniques

#### 3.5.1. Gastropexy Techniques

Two types of gastropexy techniques were performed on dogs that were included in the study: belt loop and incisional gastropexy. The gastropexy technique used is known for 114/130 patients. Forty-nine incisional (43%; 49/114) and sixty-four belt loop gastropexies (56%; 64/114) were performed.

**Figure 2:**
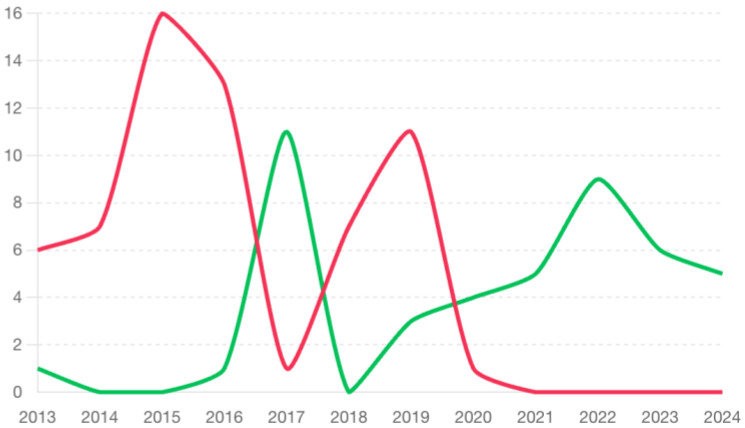
Trend of surgical techniques for gastropexy over the years. Red indicates belt loop gastropexy green represents incisional gastropexy.

The graph in Figure 2 shows the trend of the use of the two different techniques during the study period. The belt loop technique has become increasingly less used, in favor of incisional gastropexy, and was completely abandoned as of 2021.

The chi-square test that was performed revealed no statistically significant differences between the two techniques in relation to the prognosis (survival: *p*-value 0.445 and post-operative complications: *p*-value 0.61).

#### 3.5.2. Gastrectomy and Splenectomy

Other procedures performed in the treatment of patients with GDV included splenectomy and the removal of necrotic portions of the stomach, through gastric invagination or gastrectomy. This data were was obtained from the clinical records of 123 patients. Results are as follows: splenectomy was performed in 46/123 dogs (37.4%); gastrectomy was undertaken in 8/123 dogs (6.5%); and gastric invagination was performed in 10/123 dogs (8.1%).

Survival rates in dogs that had and had not underwent a splenectomy performed at the time of GDV surgery were 86.9% (40 of 46) and 98.8% (1 of 77), respectively. The chi-square test shows no correlation between gastrectomy or splenectomy and mortality (gastrectomy *p*-value 1.0; splenectomy *p*-value 0.074). All dogs treated via gastrectomy or gastric invagination survived.

Gastric invagination has not been performed since 2015.

### 3.6. Medical Therapy

Data about drugs administered after the surgery were collected from the clinical records, which were available for 90 out of 130 patients. Data about drugs and medications used in the intraoperative period were retrieved from the anesthesiologic forms, available for 37 patients. To facilitate data analysis, the drugs and medications used were grouped into the following categories:

- colloids, anti-hypotensives, and blood products;
- antibiotics;
- analgesics;
- antiarrhythmics;
- other drugs not included in the above categories.

#### 3.6.1. Colloids, Anti-Hypotensives, and Blood Products

In cases where blood pressure values required therapeutic intervention from the preoperative period to patient discharge, treatment involved fluid therapy combined with hypertensive agents. In the analyzed cases, colloidal fluid therapy was used almost exclusively during the intraoperative period and was reported in 7 out of 37 cases. In four cases, either in combination with colloids or as a replacement, hypertensive agents such as ephedrine (50 μg/kg), dobutamine (0.5 mg/kg/min), and norepinephrine (0.3 mg/kg/min) were used to restore normal arterial pressure. During the intraoperative period, blood products were used in 3 out of 37 cases to counteract surgical hemorrhages and restore normal hematocrit values.

#### 3.6.2. Antibiotics

Prophylactic antibiotic treatment is commonly administered to GDV patients. The active ingredients used in this study are listed in Figure S2 in the Supplementary Materials, with their respective frequencies.

In several cases, some antibiotics were used in combination:

- Amoxicillin and clavulanic acid + metronidazole;
- Amoxicillin and clavulanic acid + marbofloxacin;
- Amoxicillin and clavulanic acid + cefazolin;
- Cefazolin + metronidazole;
- Cefazolin + marbofloxacin.

Chronological analysis revealed an increasing trend in the use of cefazolin starting in 2018, accompanied by a gradual decline in the use of amoxicillin and clavulanic acid.

#### 3.6.3. Analgesics

The following pain medications were used in the analyzed cases:

- Methadone: 89 cases
- Buprenorphine: 79 cases
- Fentanyl: 1 case

Methadone and buprenorphine were often used in combination, with methadone primarily administered during the first 24–48 h postoperatively, followed by buprenorphine. Chronologically, the use of buprenorphine has significantly decreased in recent years, while methadone use has remained consistent in nearly all cases (89 out of 90).

#### 3.6.4. Antiarrhythmics

Given the high risk of arrhythmias in GDV patients, antiarrhythmic drugs were frequently employed. Lidocaine 2% was administered as a continuous rate infusion (CRI) at a dosage of 50 μg/kg/min, sometimes starting intraoperatively if premature ventricular contractions were present. It was used in 49 out of 90 cases (53%).

#### 3.6.5. Other Medications

During hospitalization, patients were treated with both symptomatic and prophylactic medical therapy. The most used drug was omeprazole, administered in 76 out of 90 cases. Another gastroprotectant occasionally used in conjunction with omeprazole was sucralfate (six cases). The widespread use of gastric mucosal protectants is attributed to the damage sustained by the mucosa due to hypoperfusion and potential ischemic injury, as well as the use of non-steroidal anti-inflammatory drugs (NSAIDs) in the postoperative period.

NSAIDs represent the second most used drug category. Among the active ingredients administered, meloxicam was given to 34 out of 90 patients, while carprofen was administered to 18 out of 90 patients. Following in order of usage frequency was maropitant, a central antiemetic, used in 33 out of 90 cases. Lastly, the least frequently used drug was metoclopramide, an antiemetic and prokinetic agent, administered in 4 out of 90 cases.

### 3.7. Complications

The data that were collected allowed for the identification of complications that occurred intraoperatively and during hospitalization. Information on complications occurring post-discharge were obtained from questionnaires administered to the owners.

Overall complications rate was 66/130 (50.8%), belt loop gastropexy complications rate was 31/64 (48.4%), and incisional gastropexy was 23/49 (46.9%).

Complications were categorized into three groups: intraoperative, postoperative, and post-discharge complications.

#### 3.7.1. Intraoperative Complications

These were available in 90 cases, reported in Table 2. In several cases these complications were present simultaneously.

#### 3.7.2. Complications During Hospitalization

These were available for 88 cases, reported in Table 3:

#### 3.7.3. Post-Discharge Complications

Those were available for 53 cases and are reported in Table 4.

In three of the four melena episodes, a gastric invagination for necrotic stomach segments was performed.

All the reported post-discharge complications occurred within a year of the GDV episode. Among the three patients who developed melena after gastric invagination, two required a second surgical procedure (gastrectomy) to remove the previously invaginated stomach portion. These patients recovered completely from surgery. Melena was not more evident after three days post-surgery. Suture dehiscences were surgically managed with wound curettage and suture apposition. After treatment, no more wound-related complications were observed. Episodes of vomiting were successfully addressed through symptomatic medical therapy. Five dogs had a new episode of gastric dilatation; four had been successfully decompressed; and a gastric volvulus was radiographically diagnosed in the last one. The owner refused a second surgery, and euthanasia was performed.

Owners did not report a deterioration in the quality of life of patients following surgery despite complications.

### 3.8. Survival and Hospitalization Time

Survival data were available in 118/130 dogs. Sixteen deaths related to GDV were recorded. Fifteen occurred during surgery or in the immediate postoperative period, while one patient died more than six months after admission due to a GDV recurrence. A belt-loop gastropexy was undertaken the first time.

Eight patients died during surgery: six were euthanized due to the extensive gastric necrotic areas, making their surgical removal incompatible with life. Two patients died from cardiac arrest. Seven patients died during the recovery period. Of these, two were euthanized, due to their critical condition and in agreement with their owners, while five died from spontaneous cardiac arrest.

The hospitalization time was retrieved based on patients’ medical records, as they are filled out daily until discharge. Patients who died during surgery or hospitalization were excluded from this analysis. Mean hospitalization time was 3.76 days (SD: 1.12 days; range: 2–8 days).

### 3.9. Prognostic Factors

Lactate values at presentation were reviewed. The correlation between mortality and lactate levels at presentation was analyzed. Unfortunately lactate levels were available in 26% (34/130) of dogs only.

“Normal” and “elevated” lactate levels groups were compared and the following results obtained:

- Chi-square (χ^2^): 0.935;
- Degrees of freedom: 1;
- Expected frequencies:
  ○ For “elevated lactate levels” and “death not related to GDV”: 21.41;
  ○ For “elevated lactate levels” and “death related to GDV”: 4.59;
  ○ For “normal lactate levels” and “death not related to GDV”: 6.59;
  ○ For “normal lactate levels” and “death related to GDV”: 1.41.

There is no statistically significant difference between the lactate categories examined and the mortality of the patients (*p*-value 0.334).

## 4. Discussion

### 4.1. Signalment

The study confirms that large and giant breeds are more predisposed to GDV, with a significant percentage of mixed-breed dogs (23%). However, the German Shepherd, the most frequently observed breed (33% among purebreds), is not typically considered at high risk for GDV [12], while in other studies the German Shepherd remains the predominant breed [13]. In contrast, the Great Dane, the third most common breed in this study, is recognized as high-risk in the literature [1]. This discrepancy might be due to the limited sample size or the geographic distribution of breeds. Intact males are most frequently affected by GDV, accounting for over 50% of cases, suggesting that neutering may reduce the risk [14]. This finding highlights the need for further research about the role of hormones and behaviors in GDV predisposition. The most numerous age range was between 5 and 10 years, in agreement with the results described in literature, and young dogs (under 5 years old) seem to be less frequently affected [2]. Data about body weight, especially for the range between 30 and 40 kg, confirm that weight is a significant risk factor for GDV as reported in [12]. However, population body condition score (BCS) is not available at present, indeed we cannot correlate GDV to the nutrition status. Smaller dog breeds can also develop the condition, albeit less frequently [12]. In our population two Shih-tzu were treated for this condition, with this breed having been already described in previous studies [15].

### 4.2. Anamnesis

Many dogs with GDV were already affected by other comorbidities. These data might suggest that the presence of other pathologies could be a risk factor for the development of GDV, even if, due to the small sample size, a correlation with the outcome cannot be evaluated. In the present population, 21% of dogs reported chronic gastrointestinal conditions. Some of the reported issues, such as gastritis, frequent diarrhea, and food allergies, overlap with what is reported in the literature [16,17]. It is also possible to hypothesize that hypothyroidism may indirectly contribute to GDV pathogenesis, though scientific evidence supporting a direct correlation between the two conditions is limited [18]. Hypothyroidism, by reducing the basal metabolism, leads to consequences that might favor the development of GDV syndrome. Among these, the tendency to obesity represents a potential risk factor: excess weight can negatively affect gastric motility and increase the likelihood of gastric dilation. Other aspects to consider include lethargy and reduced physical activity, which often characterize dogs with hypothyroidism [19]. Additionally, the decrease in gastric motility may have played a role in the recovery of gastrointestinal tract function and worsened complications such as the occurrence of vomiting episodes, for example, due to delayed gastric emptying.

Previous cardiac pathologies do not seem to be related to the onset of GDV, but they may have affected survival or the development of complications (such as arrhythmias) in the affected patients, worsening the myocardial distress associated with pre-existing ischemic damage.

Analysis of dietary habits shows that most owners provided their dogs with a dry food diet at the time of GDV onset, while meal distribution throughout the day is mostly uniform, with a prevalence of two meals per day. This trend aligns with previous studies reporting that the ingestion of dry food, especially in large quantities during a single meal, could represent a risk factor [20,21].

An interesting finding is that six owners reported that their dogs had drunk large amounts of water before the onset of GDV. This could suggest that excessive water intake, not necessarily corresponding to mealtimes, could be a triggering factor. Further studies are needed to establish if and how water intake could represent a significant risk, as already reported in Glickman et al. [20]. In any case, it may be useful to raise awareness among owners about this possibility so as to improve the prevention of this condition.

### 4.3. Time Distribution

During the study period (2011–2024), no significant fluctuations in the frequency of cases were observed. This suggests that the occurrence of GDV is not influenced by factors that change year by year, such as weather conditions, lifestyle trends of the owners or diagnostic capabilities. Furthermore, no significant correlation was found between the seasons and the incidence of GDV. It can be concluded that seasonal factors such as temperature, humidity, or the reproductive cycle of animals do not seem to influence the onset of the condition in a significant way. The absence of significant temporal distribution implies that the incidence of GDV is relatively constant over time, without predictable peaks. This may make the adoption of preventive measures, such as awareness campaigns or monitoring, more difficult, as the condition necessitates a high level of attention from owners throughout the entire calendar year.

### 4.4. Patient Presentation

The higher concentration of nighttime visits (49%) to the emergency service suggests that GDV may occur and manifest more frequently at night. This phenomenon could be due to the concentration of larger meals or the habit of taking longer walks in the evening, possibly due to the owners’ work commitments or to avoid the hotter hours in the summer months. A second plausible hypothesis is that owners are more likely to notice the symptoms of GDV during the evening and nighttime, when the animals are under strict observation. Analysis of the time elapsed from the onset of symptoms to hospitalization shows that most patients are referred to the emergency service within three hours. This is a positive finding, as a timely diagnosis and treatment are crucial for improving the prognosis of GDV, as reported by Beck et al. [6]. However, the presence of cases where hospitalization occurred more than 24 h after the appearance of symptoms raises concerns. At the same time, it is not possible to state that it is a definite risk factor, as these patients survived. Moreover, several studies report the absence of a correlation between poor prognosis and the increase in the time interval between symptom detection and treatment [10,22]. The delay may be due to various factors, including a poor perception of the severity of symptoms by the owners, possible logistical difficulties in reaching the emergency service, delay in identifying symptoms in outdoor dogs, or lack of awareness about the need for immediate intervention. Data from the patients’ medical records also show that the clinical status of patients upon arrival at the VTH is quite variable: from alert and reactive patients to those in uncompensated shock with hemodynamically unstable conditions. Patients presenting in shock may have been brought to the emergency service later or may have experienced a more rapid and aggressive pathological course. In contrast, patients in better conditions may have benefited from a quicker response by the owners or suffered fewer systemic repercussions from gastric torsion.

### 4.5. Surgical Techniques

The analysis of the evolution of gastropexy techniques showed a change in the approach used for the prevention of GDV recurrence. Until 2017, the belt loop gastropexy technique was the most commonly used by surgeons at this time, but from then on, a shift toward incisional gastropexy was observed. The gradual abandonment of the belt loop technique and the adoption of the incisional technique, which has become the exclusive method since 2021, suggests that surgeons have found incisional gastropexy to be an easier and faster method for preventing GDV recurrence. However, despite the change in surgical techniques (belt loop vs. incisional gastropexy), no significant differences in terms of outcome, survival, and recurrence have been observed. These results are supported by the current literature, in which there are no statistically significant differences in survival rates or recurrence of GDV between the two gastropexies [13]. Furthermore, incisional gastropexy has been described to be effective, without GDV recurrences, and to be an easy technique to perform [1,23].

The performance of splenectomies and gastrectomies did not affect the patients’ prognosis, although it is reported that, especially when combined, they are related to a negative prognosis [6,13,24,25]. The differences observed in the study results compared with those reported in the literature are likely due to the limited sample size of dogs that died as a result of GDV (15 dogs). Additionally, eight deaths occurred during the intraoperative period, prior to performing any surgical procedure beyond an exploratory celiotomy. Seven dogs died postoperatively; among them, six dogs had a splenectomy and none a gastrectomy. Therefore, in our study splenectomy did not affect the survival time (*p*-value = 0.074).

Gastric invagination is a quick and less technically demanding technique, with a lower risk of intraoperative contamination when compared with gastrectomy [26,27]. However, it has already been associated with the presence of gastric ulcers and the need for surgical revisions [27]. For these reasons, this technique was abandoned.

### 4.6. Medical Therapy

Management of blood pressure in GDV patients has been crucial, especially during the intraoperative phase. The use of colloids and medications such as ephedrine, dobutamine, and norepinephrine in dogs with severe hypotension reflects the need to restore tissue perfusion and blood volume during surgery. The use of red blood cells in a small number of cases suggests that intraoperative bleedings were rare. These results reflect what is reported in the literature, as hemorrhage development is uncommon and, when it does occur, is related to splenic parenchyma rupture due to the torsion, advanced ischemic necrosis, or avulsion of short gastric arteries [17].

Prophylactic antibiotic therapy plays an essential role in GDV management, given the high likelihood of bacterial translocation and septicemia due to gastric wall necrosis. The use of a single antibiotic or a therapy involving two active principles depends on the severity of the individual case and factors such as gastric rupture or necrosis [28]. The combination of antibiotics, such as amoxicillin and clavulanic acid with metronidazole or cefazolin, suggests a targeted approach to cover a broad spectrum of bacteria, including gram-negative bacteria, which appear to be the most commonly detected bacteria in blood cultures from patients affected by GDV [29].

Postoperative pain management has also been a priority, given the intensity of pain associated with GDV and surgery [1]. The use of methadone in the first 24–48 h postoperatively, followed by buprenorphine, indicates the presence of a structured protocol for managing acute pain and recovery. The use of lidocaine as an antiarrhythmic in more than half of the cases (53%) highlights the high risk of arrhythmias in GDV patients, often related to surgical stress, electrolyte imbalances, and hypoxic–ischemic myocardial states, even starting from the preoperative period. Analyzing the data chronologically, a change in the therapeutic protocol was observed, indicating a preference for meloxicam over carprofen among the anti-inflammatory drugs. Furthermore, the use of maropitant, which was initially administered sporadically, became consistent starting from 2018 onwards. In summary, the analysis of medical treatments for GDV patients highlights a therapeutic approach that aims to be increasingly effective, based on clinical evidence and constant monitoring of the patient’s conditions, in order to optimize recovery chances and reduce postoperative complications.

### 4.7. Complications

The most common complications observed during hospitalization were arrhythmias and the presence of abdominal effusion, with a frequency of 30% and 24%, respectively. These complications are well documented in the literature as common events in patients with GDV, often related to severe hemodynamic impairment and tissue necrosis that can occur during gastric torsion [11,30].

Hypotension, although less frequent, was observed in 7% of cases and represents a serious complication requiring immediate treatment to avoid further damage to vital organs.

After discharge, the most frequently reported complications by owners were episodes of vomiting and gastric dilatation. These issues could indicate residual gastrointestinal dysfunction or side effects from medical therapy. The development of melena, infection at the surgical site, or suture dehiscence indicates that, despite the initial success of surgery, patients may still face serious complications postoperatively, which require continuous monitoring and, in some cases, a second surgical intervention.

### 4.8. Survival and Hospitalization Time

The survival rate recorded in the sample of GDV patients was 86.4% (102/118), with 15 deaths occurring during surgery or in the immediate postoperative period. One dog died after one year due to a GDV recurrence. These data are in line with other studies that report mortality rates ranging from 10% to 23%, depending on the severity of the condition at presentation and the complications during and after surgery [1,6,9]. The observed mortality rate highlights the severity of the GDV syndrome and the importance of prompt surgical intervention and intensive pre- and postoperative management to improve prognosis. It is noteworthy that the postoperative deaths primarily resulted from spontaneous circulatory arrest, suggesting that, despite the surgery, some patients may suffer from irreversible damage to vital organs or systemic complications that cannot always be prevented or treated successfully. These data sketch out the need for the postoperative monitoring of vital parameters, with particular attention to cardiovascular parameters.

### 4.9. Prognostic Factors

The prognostic value of lactate measurements in cases of GDV has not been proved in all studies, and comparisons between studies are challenging due to variations in the treatment protocols and the timing of lactate measurements [31]. However, substantial evidence indicates that lower lactate levels at presentation, as well as a reduction in hyperlactatemia after treatment, are associated with decreased morbidity and mortality [9,32,33].

In our study the evaluation of lactate levels at presentation showed no statistically significant correlation between lactate levels and mortality in GDV patients. However, the low percentage of lactate data completeness (26.15%) may have influenced the statistical power of the study, making it impossible to draw a definitive conclusion. It has not been possible to retrieve all of the lactate measurements due to the fact that they were recorded on paper and have not been transferred to a digital database, making most of them unavailable for evaluation.

Other prognostic markers, such as inflammatory cytokines, troponin, and myoglobin, were not taken into account in this study as their evaluation was not included in the diagnostic protocols due to the need for specific test and protocols, not available in VTH ES, with which to assess these values. Future studies could benefit from more complete data collection and a deeper analysis of the abovementioned potential markers that are not reported in this study but which may provide valuable insights into the severity of the condition and prognosis, as reported in [4,5].

### 4.10. Limits of the Study

This study has several limitations that may influence the interpretation of the results. Firstly, the retrospective nature of the data collection relies on existing clinical records, which are not always complete or consistent. Furthermore, the sample only includes dogs treated at a single facility, which may not fully reflect the general canine population. Regional differences in breed prevalence, owner preferences, and the clinical management of GDV patients may influence the interpretation of the data.

It is important to highlight that the conducted studies exclusively involve patients who underwent surgery and survived long enough to reach the operating room. This limitation introduces a bias in interpreting the data, particularly regarding mortality rates and disease incidence, as it excludes data on patients affected by GDV who did not survive to this stage.

## 5. Conclusions

Medical history highlighted that pre-existing conditions, especially gastrointestinal disorders, may contribute to the risk of GDV. Diets based on dry food or the ingestion of large amounts of liquids could be triggering factors. The importance of rapid diagnosis enabling timely management of GDV is reported in the existing literature; however, the time elapsed between the detection of symptoms and medical treatment was not found to be significantly associated with the patients’ prognosis.

Incisional gastropexy is safe, fast, and effective at preventing recurrence. However, in contrast with that reported in the literature, gastrectomy and splenectomy did not influence the prognosis. The complications that required a second surgery were suture dehiscence and post-invagination gastric ulcers. The observed mortality rate is consistent with the literature. In this study, lactate levels did not prove to be a prognostic factor, as data were unavailable in the majority of cases.

## Figures and Tables

**Table 1:** Incidence of clinical signs at presentation obtained from ES medical report.

Examined Parameter	Detected Alteration	Frequency of the Alteration	Relative Percentage
Respiratory rate	Polypnea	64 out of 98	65.3%
Heart rate	Tachycardia	43 out of 98	43.8%
Heart rhythm	Arrhythmias	5 out of 83	6%
Body temperature	Hyperthermia (>39 °C)	22 out of 95	23.2%
Body temperature	Hypothermia (<37.5 °C)	13 out of 95	13.7%
Mucous membranes	Congested	40 out of 78	51.3%
Mucous membranes	Pale	11 out of 78	14.1%
State of consciousness	Depressed	16 out of 51	31.4%
Hemodynamic status	Shocked	12 out of 47	25.5%
Blood pressure	Hypotension	12out of 47	25.5%
Abdominal effusion	Presence	12 out of 78	15.4%

**Table 2:** Intraoperative complications.

Arrhythmias	10 out of 90 cases	11.10%
Hypotension	8 out of 90 cases	8.90%
Euthanasia/death	8 out of 90 cases	8.90%

**Table 3:** Complications during hospitalization. SBP: systolic blood pressure.

Abdominal effusion	21 out of 88 cases	24%
Arrhythmias	26 out of 88 cases	30%
Hypotension	5 out of 88 cases	6%
(SBP < 90 mmHg)		

**Table 4:** Post-discharge complications.

Vomiting episodes	13 out of 53 cases	25%
Melena	4 out of 53 cases	8%
Surgical site infections or suture dehiscence	4 out of 53 cases	8%
Gastric dilatation episodes	5 cases	9%

## Data Availability

The authors declare that the data found in this paper are accessible and available.

## Supplementary Materials

The following supporting information can be downloaded at https://www.mdpi.com/article/10.3390/ani15040579/s1, File S1: Owners form; Figure S1: Temporal distribution; Figure S2: Antibiotics.
