# Supplementary material for: Gastric Dilatation-Volvulus in Dogs: Analysis of 130 Cases in a Single Institution
Source: Animals (Basel). 2025 Feb 18;15(4):579. doi: 10.3390/ani15040579 (PMC11851494; doi:10.3390/ani15040579)
Supplement: Supplementary file 1 [file animals-15-00579-s001.zip › File S1 Owners form.pdf]

Dog's GDV Episode Information Form

Section 1: General Information

1. **Dog's Name:** ________________________________
2. **Breed:** ________________________________
3. **Dog's Age at the Time of the GDV Episode:** ________________________________
4. **Dog's Sex:**
   - ☐ Male
   - ☐ Female
5. **Is your dog spayed/neutered?**
   - ☐ Yes
   - ☐ No
6. **Dog's Weight:** ________________________________
7. **What type of diet did your dog have before the GDV episode?**
   - ☐ Dry
   - ☐ Wet
   - ☐ Mixed/Homemade

Section 2: Medical History

8. **Did your dog have any other significant health problems prior to the GDV episode?**
   - ☐ Yes
   - ☐ No

   If yes, which ones? ____________________________________
9. **Did your dog undergo any surgeries prior to the GDV episode?**
   - ☐ Yes
   - ☐ No

   If yes, for what reason? ____________________________________

Section 3: GDV Episode

10. **When did the GDV episode occur? (Approximate date):** ________________________________
11. **What were the first symptoms you noticed?**
    - ☐ Abdominal bloating
    - ☐ Non-productive vomiting
    - ☐ Restlessness
    - ☐ Labored breathing
    - ☐ Other (please specify): ________________________________
12. **How much time passed between the onset of symptoms and arrival at the emergency clinic?**

13. **Did your dog experience any complications during or after the procedure?**
    o ☐ Yes
    o ☐ No
    If yes, which ones? ________________________________

Section 4: Post-Episode Management

14. **How was the recovery period for your dog after the GDV episode?**

---

15. **Did you change your dog's diet after the GDV episode?**
    o ☐ Yes
    o ☐ No
    If yes, what changes did you make? ________________________________
16. **Have you taken any preventive measures to avoid a future GDV episode?**
    o ☐ Yes
    o ☐ No
    If yes, what measures? ________________________________
