# Supplementary figures and images for: Gastric Dilatation-Volvulus in Dogs: Analysis of 130 Cases in a Single Institution
Source: Animals (Basel). 2025 Feb 18;15(4):579. doi: 10.3390/ani15040579 (PMC11851494; doi:10.3390/ani15040579)

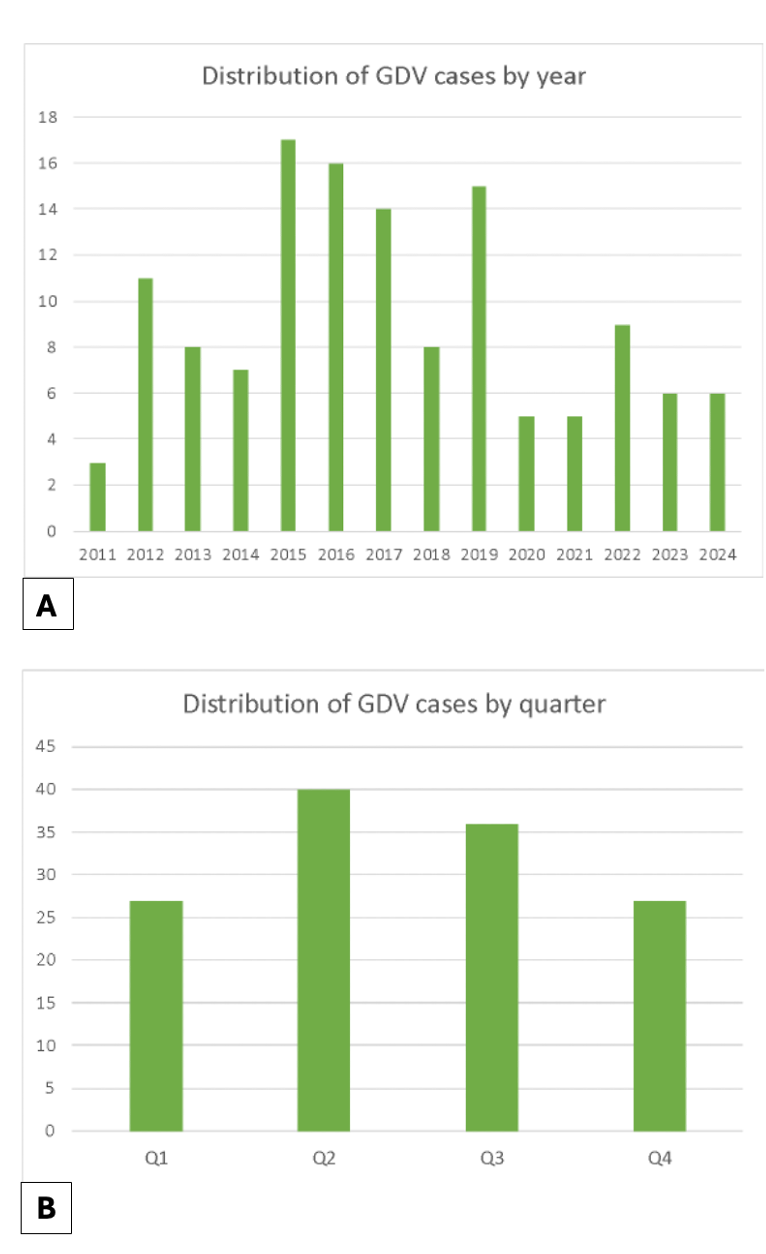

Supplement: Supplementary file 1 [file animals-15-00579-s001.zip › Figure S1 temporal distribution.png]

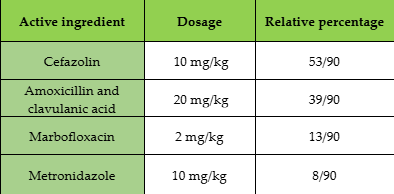

Supplement: Supplementary file 1 [file animals-15-00579-s001.zip › Figure S2.png]
